# Specialized Roles of Human Natural Killer Cell Subsets in Kidney Transplant Rejection

**DOI:** 10.3389/fimmu.2019.01877

**Published:** 2019-08-07

**Authors:** Katrina Kildey, Ross S. Francis, Sebastian Hultin, Michelle Harfield, Kurt Giuliani, Becker M. P. Law, Xiangju Wang, Emily J. See, George John, Jacobus Ungerer, Ray Wilkinson, Andrew J. Kassianos, Helen Healy

**Affiliations:** ^1^Conjoint Internal Medicine Laboratory, Chemical Pathology, Pathology Queensland, Brisbane, QLD, Australia; ^2^Kidney Health Service, Royal Brisbane and Women's Hospital, Brisbane, QLD, Australia; ^3^Princess Alexandra Hospital, Brisbane, QLD, Australia; ^4^Medical School, University of Queensland, Brisbane, QLD, Australia; ^5^Institute of Health and Biomedical Innovation, Queensland University of Technology, Brisbane, QLD, Australia; ^6^School of Biomedical Sciences, Queensland University of Technology, Brisbane, QLD, Australia

**Keywords:** natural killer cells, innate lymphocytes, kidney allograft rejection, T cell mediated rejection, antibody-mediated rejection

## Abstract

**Background:** Human natural killer (NK) cells are key functional players in kidney transplant rejection. However, the respective contributions of the two functionally distinct human NK cell subsets (CD56^bright^ cytokine-producing vs. CD56^dim^ cytotoxic effector) in episodes of allograft rejection remain uncertain, with current immunohistochemical methods unable to differentiate these discrete populations. We report the outcomes of an innovative multi-color flow cytometric-based approach to unequivocally define and evaluate NK cell subsets in human kidney allograft rejection.

**Methods:** We extracted renal lymphocytes from human kidney transplant biopsies. NK cell subsets were identified, enumerated, and phenotyped by multi-color flow cytometry. Dissociation supernatants were harvested and levels of soluble proteins were determined using a multiplex bead-based assay. Results were correlated with the histopathological patterns in biopsies—no rejection, borderline cellular rejection, T cell-mediated rejection (TCMR), and antibody-mediated rejection (AMR).

**Results:** Absolute numbers of only CD56^bright^ NK cells were significantly elevated in TCMR biopsies. In contrast, both CD56^bright^ and CD56^dim^ NK cell numbers were significantly increased in biopsies with histopathological evidence of AMR. Notably, expression of the activation marker CD69 was only significantly elevated on CD56^dim^ NK cells in AMR biopsies compared with no rejection biopsies, indicative of a pathogenic phenotype for this cytotoxic NK cell subset. In line with this, we detected significantly elevated levels of cytotoxic effector molecules (perforin, granzyme A, and granulysin) in the dissociation supernatants of biopsies with a histopathological pattern of AMR.

**Conclusions:** Our results indicate that human NK cell subsets are differentially recruited and activated during distinct types of rejection, suggestive of specialized functional roles.

## Introduction

Kidney transplantation is the gold standard treatment for end stage kidney disease, with superior quality of life and patient survival compared to dialysis. Despite advances in kidney transplantation techniques and immunosuppression therapy, immunological rejection continues to account for loss of graft function and eventually graft loss (1). Immune-mediated allograft rejection is classified histopathologically into two types: T cell-mediated rejection (TCMR) and antibody-mediated rejection (AMR) (2, 3). TCMR is characterized by tubulointerstitial inflammation mediated by host alloreactive lymphocytes targeting donor human leukocyte antigen (HLA) molecules in the graft, whilst AMR is a process of microvascular inflammation (glomerulitis, peritubular capillaritis) driven by donor-specific antibodies (DSA) interacting with the allograft endothelium (4–6). Most immuno-biological studies of kidney allograft rejection have focused on conventional T (CD4^+^ or CD8^+^) and B cells. Less is known about the roles of innate lymphocytes in the different patterns of immune-mediated allograft rejection.

Natural killer (NK) cells are innate lymphocytes that have an immune surveillance function under homeostatic conditions, but can be rapidly recruited to sites of inflammation under pathological conditions. NK cells are activated by a combination of inhibitory and activating signals orchestrated through cell surface receptors and/or cytokine stimulation. Once activated, NK cells display immediate effector function through the production of pro-inflammatory cytokines and via cytotoxic activity (7).

Human NK cells are classically defined as CD3^−^/CD56^+^/CD335 (NKp46)^+^ mononuclear cells. They are subcategorised based on expression levels of CD56 (neural cell adhesion molecule) into low-density (CD56^dim^) and high density (CD56^bright^) subsets. The two NK cell subsets differ in phenotypic and functional properties. CD56^dim^ NK cells express high levels of CD16 (low affinity receptor for IgG, FcγRIII) (8) and are considered functionally to be cytotoxic effector cells (9). Upon activation, CD56^dim^ NK cells produce cytotoxic granules containing perforin/granulysin that, when released, create pores in the cell membranes of targeted cells and granzymes that induce apoptosis (9). In contrast, CD56^bright^ NK cells express high levels of CD56, are CD16^−/low^ and mediate immune responses by secreting pro-inflammatory cytokines [e.g., interferon (IFN)-γ] (9).

NK cells are emerging as powerful drivers in immune-mediated kidney allograft rejection. Their functional role has been established in mouse models of kidney allograft injury (10–12). Initial investigations in humans, focused primarily on peripheral blood NK cells from kidney transplant recipients and transcriptomic studies of allograft biopsies, provide compelling evidence supporting a role for NK cells in kidney allograft injury (13–18). Indeed, immunohistochemical (IHC)-based studies report significant associations between human NK cells and both TCMR (19, 20) and AMR (18, 21, 22). However, these IHC-based studies are limited to single antigen (CD56, CD16, or NKp46) labeling to identify human NK cells, a technical approach that cannot unequivocally define this innate lymphocyte population nor differentiate between the distinct NK cell subsets. Thus, to date, the discrete roles of kidney NK cell subsets in different types of human allograft rejection have not been reported.

These technical limitations can be addressed using multi-parameter staining methodologies that accurately identify, enumerate and phenotype human NK cells, in particular, NK cell subsets, in kidney allograft biopsies. In this study, we extend our previously published multi-color flow cytometry-based approach to provide, for the first time, a comprehensive mapping of human NK cell subsets in kidney allograft rejection, ascribing specialized roles during the two patterns of immune-mediated rejection (TCMR vs. AMR).

## Methods

### Study Design

Kidney transplant recipients (*n* = 56) were biopsied at the Royal Brisbane and Women's Hospital or Princess Alexandra Hospital between 2015 and 2018. All biopsies were undertaken for clinical indications. Written informed consent for participation in the study was obtained. The Royal Brisbane and Women's Hospital Human Research Ethics Committee (2006/072) and the Princess Alexandra Hospital Ethics Committee (HREC/16/QPAH/214) approved the study.

### Kidney Tissue Specimens

Fresh biopsy specimens were taken with either an 18-gauge or 16-gauge biopsy needle (Biopsybell, Mirandola, Italy) and immediately divided for (i) tissue dissociation (1–5 mm of a core biopsy specimen); and (ii) assessment of allograft rejection by specialist renal histopathologists blinded to experimental results. The biopsies were examined for rejection in the pathology departments of participating hospitals.

Samples were graded according to the Banff-classification (23). According to these criteria, biopsies were then grouped into: no evidence of rejection (no rejection), borderline cellular rejection (borderline), TCMR alone, or biopsies with an indication of AMR. Samples that arrived for processing >12 h post collection were excluded. Biopsies that had other diagnoses such as BK nephropathy, recurrent patterns of glomerulonephritis like IgA nephropathy and additional pathology (e.g., amyloidosis) were also excluded from the study.

### Tissue Dissociation for Flow Cytometric Analysis

Allograft biopsy specimens excess to clinical diagnostic need were digested within 12 h of collection using our published protocol (24). In brief, kidney cortical tissue was digested with 1 mg/ml collagenase P (Roche, Mannheim, Germany) in the presence of 20 mg/ml DNase I (Roche) for 15 min. Following centrifugation, supernatant was collected for assessment of soluble cytotoxic effector proteins. Tissue was further digested with 10 mg/ml trypsin + 4 mg/ml ethylenediamine tetraacetic acid (EDTA) (Life Technologies, Grand Island, NY) for 10 min.

### Flow Cytometry

Single cell suspensions were initially stained with LIVE/DEAD Fixable Near-IR Dead Cell Stain Kit (Life Technologies) to exclude non-viable cells. Cells were then incubated with Human TruStain FcX Blocking Solution (Biolegend, San Diego, CA) at room temperature for 5 min and then stained on ice for 30 min with combinations of test- (0.25 μg per antibody) (Table 1) or isotype-matched control antibodies in cold fluorescence-activated cell sorter buffer (0.5% bovine serum albumin [Sigma-Aldrich, St. Louis, MO] and 0.02% sodium azide [Sigma-Aldrich] in phosphate buffered saline).

**Table 1:** Antibodies used for flow cytometric staining.

**Antigen**	**Clone**	**Flurophore**	**Source**
CD45	HI30	BV510	BioLegend
CD14	M5E2	AF700	BioLegend
CD3	OKT3	BV650	BioLegend
CD19	HIB19	FITC	BD
CD56	HCD56	PerCPCy5.5	BioLegend
CD16	3G8	PE-CF594	BD
HLA-DR	L243	BV785	BioLegend
CD69	FN50	PE	BD

**Figure 1:**
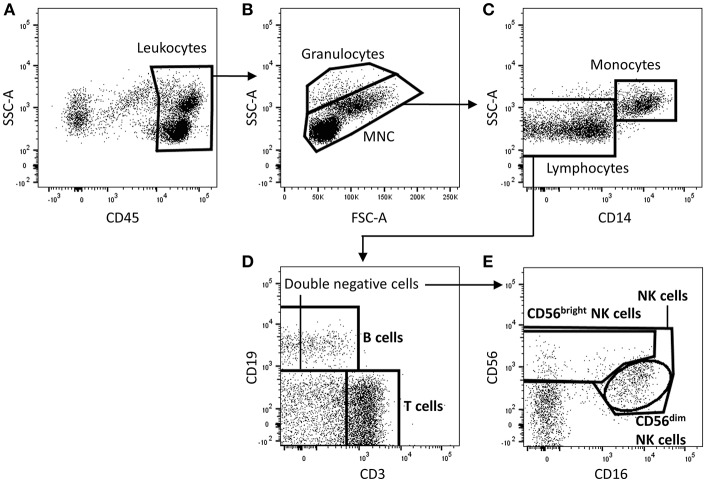
Identification of T cells, B cells, and natural killer (NK) cell subsets in human kidney tissue. Gating strategy used to identify T cells (CD3^+^), B cells (CD19^+^), total NK cells (CD3^−^ CD19^−^ CD56^+^ lymphocytes), and NK cell subpopulations (CD56^dim^ and CD56^bright^ NK cells) in human kidney transplant tissue. Single, live, CD45^+^ mononuclear cells (MNC) and granulocytes are gated on a forward-scatter (FSC)/side-scatter (SSC) plot **(A,B)**. Total lymphocytes are distinguished from granulocytes and monocytes based on low SSC and absent CD14 expression **(C)**. Total lymphocytes are further separated into T cells or B cells by their expression of CD3 and CD19 respectively **(D)**. NK subpopulations, CD56^bright^ and CD56^dim^ NK cells, are identified based on CD56 intensity and CD16 expression **(E)**. Representative flow cytometric data from 1 of 10 individual antibody-mediated rejection (AMR) renal biopsy specimens are shown. An identical gating strategy was used for no rejection, borderline rejection and T cell-mediated rejection (TCMR) biopsies. MNC, mononuclear cells; FSC-A, forward-scatter area; SSC-A, side-scatter area.

Flow-Count Fluorospheres (Beckman Coulter, Brea, CA) were used for direct determination of absolute counts following the manufacturer's recommendations. Briefly, target cell concentrations (cells/μl) were calculated as the total number of target cells counted/total number of fluorospheres counted X Flow-Count Fluorosphere concentration. This value was then multiplied by the total sample volume to obtain absolute counts for each target cell population. Total cell counts were then normalized to cell numbers per cubic centimeter of tissue, in which the volume of renal tissue was calculated as πr^2^ × length of biopsy tissue, where the radius (r) of a 16-gauge biopsy specimen is 0.8 mm and 18-gauge biopsy specimen is 0.6 mm. Cell acquisition was performed on an LSR Fortessa (BD Biosciences, San Jose, CA) and data analyzed with FlowJo software (TreeStar, Ashland, OR) to identify immune cell populations as presented in Figure 1.

### Quantification of Cytokines by Multi-analyte Flow Cytometric Immunoassay

Cytotoxic effector proteins in tissue dissociation supernatants were quantified by the LEGENDplex™ Multi-analyte flow assay kit (human CD8/NK panel) according to the manufacturer's instructions (Biolegend, San Diego, USA). Data acquisition was performed on an LSR Fortessa (BD Biosciences, San Jose, CA). Standard curve and concentrations were calculated with BioLegend's LEGENDplex™ Data Analysis Software (Biolegend, San Diego, USA). Cytokine values were normalized to pg per cm^3^ of tissue.

### Statistics

All statistical tests were performed using Prism 7.0 analysis software (GraphPad Software, La Jolla, CA). Multiple comparisons were performed using a Kruskal-Wallis test with Dunn's post-test. A Mann-Whitney *u*-test was used for comparisons of non-parametric data from two groups. *P* < 0.05 were considered statistically significant.

## Results

### Human Population Demographics

As reported in Table 2, the mean age of the 56 patients in the study was 52.2 ± 14.5 years (range 20–80 years), with 66.1% (37/56) male. The majority of patients (85.7%; 48/56) undergoing biopsy had not undergone previous transplantation, suggesting a reasonably unsensitised population. Cadaveric transplants accounted for 92.8% (52/56) of all biopsies, with donor after brain death (DBD) being the most common type of cadaveric graft biopsied. The median HLA matching was 4/6. All but one patient underwent basiliximab, mycophenolic acid, tacrolimus, and prednisolone based induction therapy, with the remaining patient receiving thymoglobulin induction. The majority of patients (55.4%; 31/56) were classed as low immune risk at transplantation with undetectable calculated Panel Reactive Antibodies (cPRA 0%), whilst highly sensitized patients with cPRA >95% accounted for only 3.6% (2/56) of total patients. The majority of patients (66.1%; 37/56) underwent biopsy within the first 3 months of transplantation. Mean creatinine at time of biopsy was 219.1 ± 106.7 μmol/L (*n* = 41), with 15 patients who were haemodialysis-dependent at time of biopsy due to delayed graft function or kidney injury excluded from mean creatinine calculations.

**Table 2:** Demographic and clinical characteristics of human kidney transplant patients in study cohort.

		**All biopsy (*n* = 56)**	**No rejection (*n* = 17)**	**Borderline (*n* = 22)**	**TCMR (*n* = 7)**	**AMR (*n* = 10)**
Mean age (years) [SD]		52.2 [14.5]	53.0 [13.4]	49.4 [14.4]	45.1 [18.2]	58.1 [7.4]
Male : female (*n*)		37:19	13:4	13:9	4:3	7:3
First transplant (*n*)		48	13	20	7	8
Transplant type	DBD (*n*)	34	10	12	5	7
	DCD (*n*)	8	2	4	2	0
	Cadaveric-NOS (*n*)	10	4	4	0	2
	Living donor (*n*)	4	1	2	0	1
Median HLA match		4	4	5	4	4
DSA present (*n*)		5	2	0	0	3
cPRA	0% (*n*)	31	11	14	3	3
	>0–95% (*n*)	4	1	2	0	1
	>95% (*n*)	2	2	0	0	0
	Unavailable	19	3	6	4	6
Biopsy within 3 m of transplantation (*n*)		37	13	14	3	7
Mean creatinine prior to biopsy (μmol/L) [SD]		219.1 [106.7]	284.9 [288.7]	222.0 [135.8]	145.0 [54.4]	267.1 [135.1]
Patients receiving haemodialysis at biopsy (*n*)		15	6	7	1	1

*DBD, donor after brain death; DCD, donor after circulatory death; Cadaveric-NOS, Cadaveric-Not otherwise specified; HLA, human leukocyte antigens; DSA, Donor-specific antibodies (pre-existing); cPRA, calculated Panel Reactive Antibodies (at transplantation)*.

The transplant biopsy specimens were categorized based upon histopathological examination by renal histopathologists blinded to experimental results. Samples were graded according to the Banff-classification (23). According to these criteria, the 56 biopsies sorted into groups without histopathological evidence of rejection (no rejection; *n* = 17; 13 males/4 females; mean age of 53.0 ± 13.4 years), borderline cellular rejection (borderline; *n* = 22; 13 males/9 females; mean age of 49.4 ± 14.4 years), T cell-mediated rejection (TCMR; *n* = 7; 4 males/3 females; mean age of 45.1 ± 18.2 years) and antibody-mediated rejection (AMR; *n* = 10; 7 males/3 females; mean age of 58.1 ± 7.4 years). Demographic and clinical characteristics of these cohorts are given in Table 2.

### Identification of NK Cell Subsets in Human Allograft Kidney Tissue

Human allograft kidney tissue was enzymatically digested to obtain single cells for flow cytometric analysis. Briefly CD45^+^ leukocytes separated into granulocytes (with higher side scatter) and mononuclear cells (Figures 1A,B). The mononuclear cells were further divided into CD14^+^ monocyte and CD14^−^ lymphocyte populations (Figure 1C). Lymphocytes were then delineated into CD3^+^ T cells, CD19^+^ B cells, and CD3^−^ CD19^−^ double-negative cells (Figure 1D). Within this double-negative population, total NK cells were identified as CD56^+^ cells, with CD56^bright^ CD16^−/low^ and CD56^dim^ CD16^+^ NK cell subsets defined for the first time in human kidney allograft tissue (Figure 1E). Importantly, these surface molecules used to identify NK cells subsets in kidney allograft tissue were resistant to proteolytic cleavage as confirmed by enzymatic digestion (with collagenase P and trypsin-EDTA) of peripheral blood mononuclear cells (data not shown).

### Significantly Increased Numbers of Total NK Cells in Allograft Biopsy Specimens With TCMR and AMR

In order to profile the major lymphocyte populations in human kidney allograft tissue, we enumerated total T (CD3^+^) cells, B (CD19^+^) cells, and NK (CD3^−^ CD19^−^ CD56^+^) cells in patient biopsies. Biopsies were stratified based on the histopathological pattern of rejection, classified as: unsuspicious biopsy (no rejection), borderline cellular rejection, TCMR or AMR.

**Figure 2:**
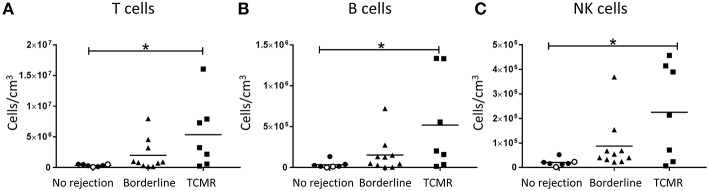
Significantly increased T cells, B cells, and NK cells in TCMR. Absolute numbers of total **(A)** T cells, **(B)** B cells, and **(C)** NK cells in kidney transplant biopsy tissue with histopathological diagnosis of no rejection (*n* = 7), borderline cellular rejection (*n* = 10), and T cell-mediated rejection (TCMR; grades I–II; *n* = 7). Values for individual donors are presented; donors with DSA are identified using open symbols; bars represent means. *P < 0.05, Kruskal-Wallis test with Dunn's post-test.

To establish the independent association between lymphocyte numbers and the severity of cellular rejection as graded using Banff criteria, we firstly performed a subgroup analysis in which patients with a histopathological pattern of AMR were excluded. For this analysis, biopsies were stratified based on the severity of cellular rejection into borderline rejection and TCMR rejection (grades I–II). Quantification using Flow-Count Fluorospheres showed numbers of total T cells, B cells, and NK cells to be significantly increased in biopsy specimens with a histopathological pattern of TCMR compared with biopsy specimens with no evidence of rejection (Figures 2A–C; *p* < 0.05).

**Figure 3:**
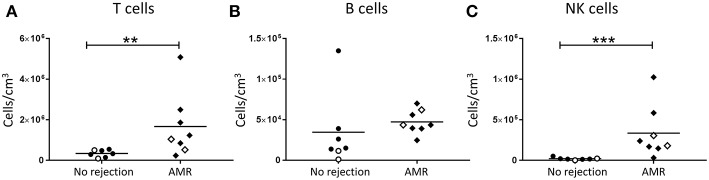
Significantly increased T cells and NK cells in AMR. Absolute numbers of total **(A)** T cells, **(B)** B cells, and **(C)** NK cells in kidney transplant biopsy tissue with histopathological diagnosis of no rejection (*n* = 7) and with indication of AMR (AMR; *n* = 8). Values for individual donors are presented; donors with DSA are identified using open symbols; bars represent means. **P < 0.01, ***P <0.001, Mann-Whitney test.

To independently assess lymphocyte numbers in association with AMR, a second subgroup analysis was performed in which patients with cellular rejection alone were excluded. Total T cell and NK cell numbers were significantly increased in biopsies with a histopathological pattern of AMR compared with no rejection (Figure 3A; *p* < 0.01 and Figure 3C; *p* < 0.001, respectively), whilst no statistical difference was observed for total B cells (Figure 3B). Collectively, these results associate human NK cells with both TCMR and AMR.

### Significantly Increased Numbers of CD56^bright^ NK Cells in Allograft Biopsy Specimens With TCMR

**Figure 4:**
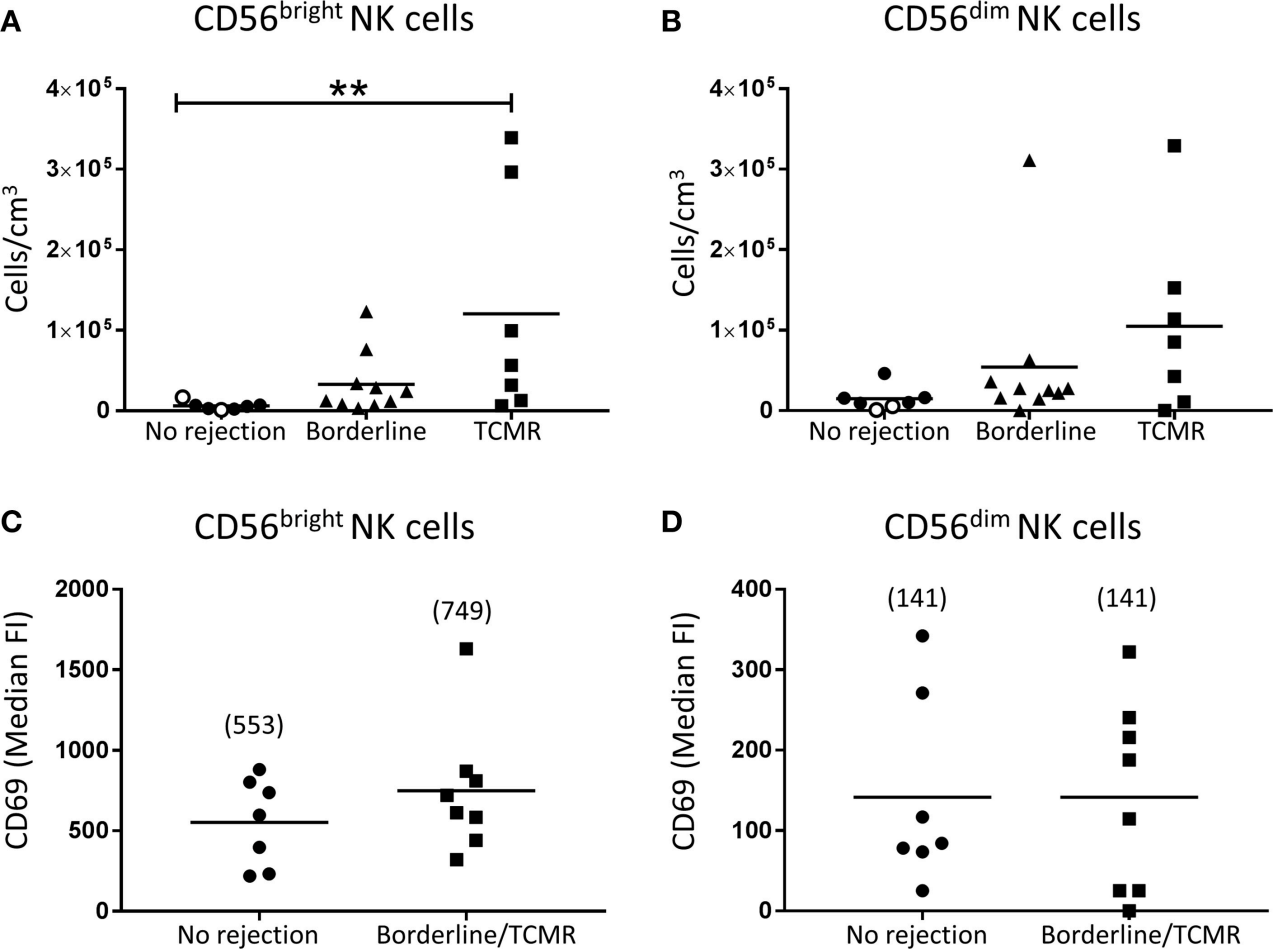
Significantly increased CD56^bright^ NK cells in TCMR. **(A,B)** Absolute numbers of total **(A)** CD56^bright^ NK cells and **(B)** CD56^dim^ NK cells in kidney transplant biopsy tissue with histopathological diagnosis of no rejection (*n* = 7), borderline cellular rejection (*n* = 10) and T cell-mediated rejection (TCMR; grades I–II; *n* = 7). Values for individual donors are presented; donors with DSA are identified using open symbols; bars represent means. **(C,D)** Surface expression of CD69 on **(C)** CD56^bright^ NK cells and **(D)** CD56^dim^ NK cells in kidney transplant biopsy tissue with histopathological diagnosis of no rejection (*n* = 7) and borderline or T cell-mediated rejection (*n* = 8). Median fluorescence intensity (median FI) values for individual donors are presented; bars represent means, with mean values presented in parentheses. **P < 0.01, Kruskal-Wallis test with Dunn's post-test.

We next assessed human kidney NK cells at a subset level. Firstly, we examined the absolute numbers of CD56^bright^ and CD56^dim^ NK cell subsets in allograft biopsies with cellular rejection alone (borderline/TCMR). Notably, only the CD56^bright^ NK cells were significantly elevated in the TCMR group compared with biopsies with no evidence of rejection (*P* < 0.01; Figures 4A,B). We also examined the phenotypes of the human kidney NK cell subsets in cellular rejection. Although not reaching statistical significance, expression levels of activation marker CD69 were elevated on CD56^bright^ NK cells in the borderline/TCMR group compared with non-rejecting biopsies (Figure 4C), whilst CD69 expression levels on CD56^dim^ NK cells were comparable between the two groups (Figure 4D).

### CD56**^dim^** NK Cells With an Activated Phenotype Associated With AMR

**Figure 5:**
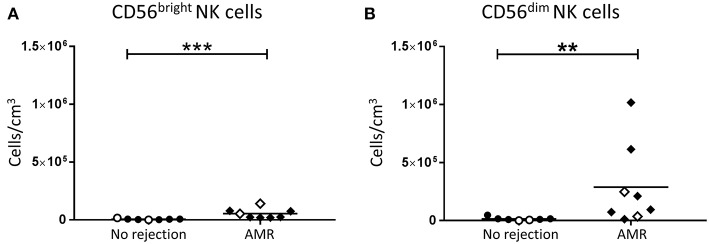
Significantly increased CD56^bright^ NK cells and CD56^dim^ NK cells in AMR. Absolute numbers of total **(A)** CD56^bright^ NK cells and **(B)** CD56^dim^ NK cells in kidney transplant biopsy tissue with histopathological diagnosis of no rejection (*n* = 7) and with indication of AMR (AMR; *n* = 8). Values for individual donors are presented; donors with DSA are identified using open symbols; bars represent means. **P < 0.01, ***P < 0.001, Mann-Whitney test.

In order to investigate the role of human NK cell subsets in humoral rejection, we examined the absolute numbers of CD56^bright^ and CD56^dim^ NK cells in allograft biopsies with a histopathological pattern of AMR. Interestingly, both CD56^bright^ (*p* < 0.001; Figure 5A) and CD56^dim^ NK cell subsets (*p* < 0.01; Figure 5B) were significantly increased in the AMR group compared with non-rejecting biopsies.

**Figure 6:**
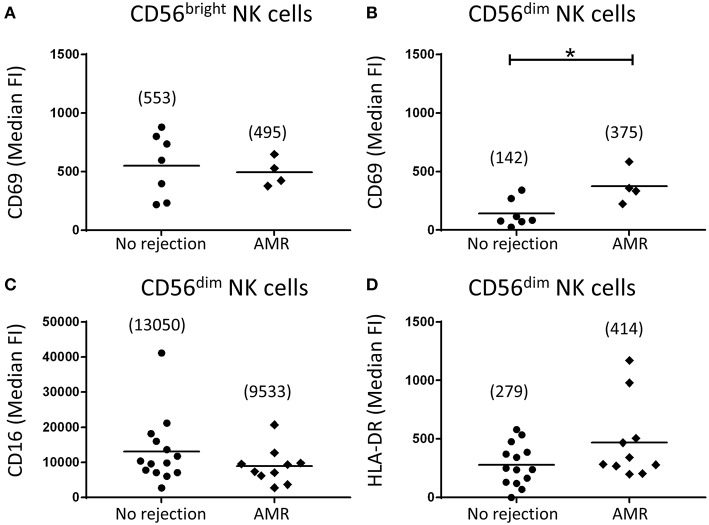
Human CD56^dim^ natural killer (NK) cells in AMR biopsies display an activated phenotype. **(A,B)** Surface expression of CD69 on CD56^bright^ NK cells **(A)** and CD56^dim^ NK cells **(B)** in biopsy specimens without (no rejection; *n* = 7) and with AMR (*n* = 4). **(C,D)** Surface expression of **(C)** CD16 and **(D)** HLA-DR on CD56^dim^ NK cells in biopsy specimens without (no rejection; *n* = 14) and with AMR (*n* = 10). Median fluorescence intensity (median FI) values for individual donors are shown; bars represent means, with mean values presented in parentheses. *P < 0.05, Mann-Whitney test.

Expression levels of activation markers on NK cell subsets were again assessed. Whilst CD69 expression levels on CD56^bright^ NK cells were similar between the AMR and no rejection groups (Figure 6A), expression of CD69 on CD56^dim^ NK cells was significantly elevated in AMR biopsies (*p* < 0.05; Figure 6B), suggestive of an activated phenotype. Human CD56^dim^ NK cells are reported to downregulate CD16 expression and upregulate HLA-DR upon activation (16). Consistent with an activated phenotype, we observed trends toward lower CD16 expression (Figure 6C) and higher HLA-DR (Figure 6D) on CD56^dim^ NK cells from biopsies with evidence of AMR; however, these did not reach statistical significance. Collectively, these results indicate that CD56^dim^ NK cells shift toward a more activated state in the AMR micro-environment.

### Significantly Elevated Levels of Cytotoxic Effector Molecules in AMR Biopsies

**Figure 7:**
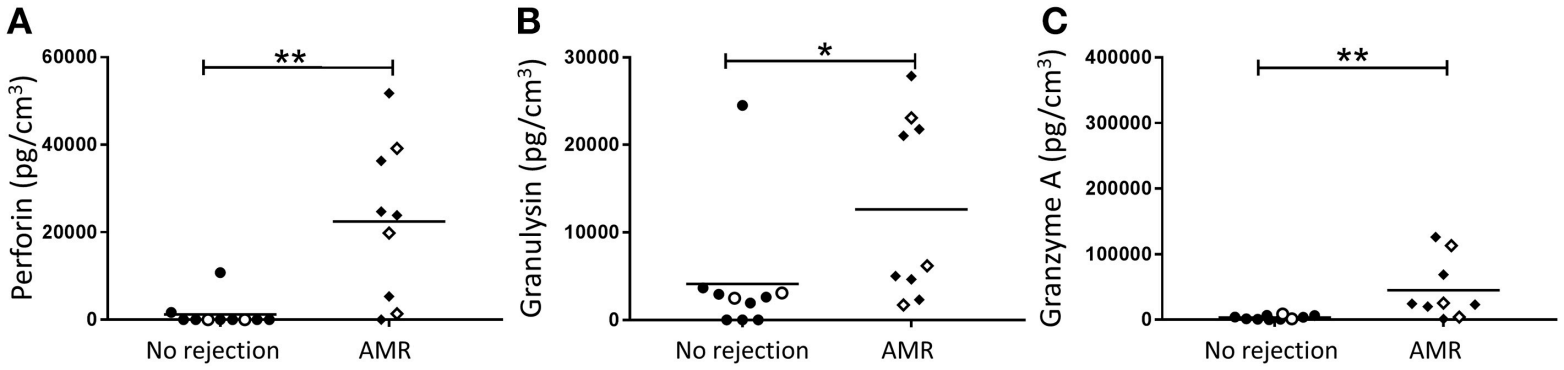
Human kidney tissue from patients with an indication of AMR have an elevated cytotoxic effector molecule profile. Expression of **(A)** Perforin, **(B)** Granulysin, and **(C)** Granzyme A in transplant biopsy specimens without (no rejection; *n* = 10) and with AMR (*n* = 9). Concentrations of effector molecules normalized to cubic centimeters of kidney tissue for individual donors are shown; donors with DSA are identified using open symbols; bars represent means. *P < 0.05, **P < 0.01, Mann-Whitney test.

The primary function of activated CD56^dim^ NK cells is cytotoxicity through the release of effector molecules perforin, granulysin, and granzymes. Thus, the supernatants from dissociated allograft biopsies were analyzed for levels of these cytotoxic effector molecules. Consistent with a putative role for activated CD56^dim^ NK cells in AMR, we observed significantly elevated levels of perforin (*p* < 0.01; Figure 7A), granulysin (*p* < 0.05; Figure 7B), and granzyme A (*p* < 0.01; Figure 7C) in biopsies with a histopathological pattern of AMR compared with non-rejecting biopsies.

## Discussion

Current treatment modalities offer non-specific therapeutic targets in the treatment of TCMR and especially AMR, and while patients can be risk stratified using donor and recipient immunological (cytotoxic and flow cross-match, HLA mismatching, DSAs, and previous recipient sensitization) and non-immunological (ischemia-reperfusion injury, warm and cold ischemic time) parameters, no biomarker exists to predict which patients will develop renal allograft rejection. To more accurately define the local drivers of immune-mediated allograft rejection, we report the use of a multi-color flow cytometric approach to analyse immune cell populations in human renal allograft tissue. Muczynski et al. used this technique to examine the relative proportions (not absolute counts) of immune cell populations in kidney allograft rejection (25). Here, we report, for the first time, the use of this methodology to examine absolute numbers of discrete lymphocyte populations (including NK cell subsets) in different patterns of immune-mediated rejection (TCMR, AMR).

To focus in on putative drivers of grades of cellular rejection, we performed a subgroup analysis in which AMR biopsies were excluded and remaining biopsies stratified into borderline cellular rejection and TCMR (grades I–II). Using this approach, we observed significantly elevated numbers of total NK cells in TCMR biopsies, consistent with previous transcriptomic and IHC-based studies of cellular rejection. An early transcriptomic investigation of kidney allograft biopsies reported associations between high NK cell transcript expression and histological patterns of TCMR (14). More recently, IHC-based studies showed significant associations between human NK cells and TCMR (19, 20, 22). In agreement with our findings, dos Santos et al. reported significantly increased numbers of CD56^+^ cells associated with histopathological manifestations of interstitial inflammation and tubulitis; both hallmark features of TCMR (20, 22). Another study reported a positive correlation between the number of CD56^+^ cells and the severity of TCMR (19). However, these IHC-based evaluations have identified NK cells based on the expression of a single marker (e.g., CD56) and thus, none of these studies are able to irrefutably exclude the interference of CD3^+^ CD56^+^ NK-like T cells or evaluate NK cell subsets.

Through the use of multi-parameter flow cytometry, our group has extended these earlier investigations to unequivocally identify and characterize human CD56^bright^ and CD56^dim^ NK cell subsets in kidney allograft tissue. Indeed, in TCMR biopsies, we observed significantly elevated numbers of only the CD56^bright^ NK cell subset (and not CD56^dim^ NK cells). CD56^bright^ NK cells function to trigger pathological immune responses through the production of pro-inflammatory cytokines (e.g., IFN-γ, TNF-α) and chemokines (e.g., monokine induced by gamma interferon; MIG) (9, 26, 27). It is thus tempting to speculate that CD56^bright^ NK cells play a specialized functional role in TCMR pathology by secreting these pro-inflammatory molecules that, in turn: (1) enhance recruitment of alloreactive T cells (28, 29) and (2) upregulate HLA alloantigens (e.g., MHC class I and II) on target donor kidney cells to make them more susceptible to cytotoxic killing (26).

AMR remains one of the major barriers to graft survival in kidney transplant patients. Diagnoses of AMR center on the detection of DSA, complement deposition and the presence of inflammatory effector cells in the peritubular capillaries and glomeruli (30). Thus, we next identified and examined the role of effector NK cells in AMR pathology. We observed significantly elevated numbers of total NK cells in biopsies with a histopathological pattern of AMR, in line with earlier studies of humoral rejection. Indeed, previous investigations using single marker immunostaining (CD56 or NKp46) reported elevated NK cell numbers associated with peritubular capillaritis (13, 19), glomerulitis (22), microcirculatory inflammation, and peritubular C4d deposition (18), associating NK cells with microcirculatory injury. Furthermore, elevated numbers of CD56^+^ cells have been shown to correlate with AMR and poor graft survival (19). Differential gene expression analyses of allograft biopsies have also demonstrated NK cell-related transcriptomic signatures in biopsies from patients with DSA and microcirculatory damage (14, 18).

We extended the work of these studies by examining human NK cells in AMR biopsies at a subset level. We report here that numbers of both CD56^bright^ and CD56^dim^ NK cells were elevated in biopsies with a histological pattern of AMR. However, only CD56^dim^ NK cells uniquely displayed significantly elevated CD69 levels in biopsy-proven AMR, suggesting this subset specifically is driven to an activated phenotype within the AMR micro-environment. Our results are in line with a recent study by Hoffman et al. showing that NK cells in the peripheral blood of kidney transplant recipients display an activated phenotype (16). In particular, this study reported the presence of activated circulating CD56^dim^ NK cells in kidney transplant recipients, characterized by up-regulated CD69 and HLA-DR, as well as reduced expression of CD16 (16). Similarly, the kidney CD56^dim^ NK cells in our AMR biopsies displayed elevated HLA-DR and reduced CD16 expression levels, although these did not reach statistical significance. Given that only this cytotoxic subset was both elevated in number and displayed an activated phenotype in AMR biopsies, our findings specifically implicate the CD16-expressing CD56^dim^ NK cell subset in the pathogenesis of AMR.

Antibody-mediated activation of NK cells via triggering of CD16 on their cell surface has been proposed to drive antibody-dependent cellular cytotoxicity (ADCC) and thus, allograft rejection (21). Indeed, gene transcript profiling of AMR biopsies has provided initial evidence for NK cell activation and signaling via engagement of Fc receptor CD16 with IgG DSA (17). Critically, in our AMR biopsies, CD56^dim^ NK cells express high levels of cell surface CD16, making them capable of engaging with DSA bound to allograft endothelial cells and, in turn, releasing cytotoxic granules (containing perforin, granulysin, and granzyme A) that can trigger targeted allograft apoptosis. In line with this concept, we found significantly elevated levels of perforin, granulysin, and granzyme A in biopsies with AMR. We speculate that CD56^dim^ NK cells contribute to the burden of cytotoxic molecules observed in biopsies with AMR. In support of our hypothesis, a recent study of the molecular signatures in human AMR biopsies strongly associated endothelial injury and cytotoxic molecules characteristic of CD56^dim^ NK cells [e.g., granulysin (*GNLY*) and *FGFBP2*] with chronic AMR (17). In conjunction with these earlier reports, our collective findings in AMR biopsies strongly support an ADCC-mediated pathogenic function for the human CD56^dim^ NK cell subset in humoral rejection.

Previous studies have reported reduced numbers of CD56^dim^ NK cells in the peripheral blood of kidney transplant recipients with indicative AMR (15, 31). In one study, the presence of DSA was associated with reduced numbers of CD56^dim^ NK cells in peripheral blood of kidney transplant recipients compared to healthy donors (31). A second, larger cohort confirmed this finding, reporting patients with anti-HLA DSA had lower proportions and absolute numbers of peripheral blood CD56^dim^ NK cells compared to patients without HLA antibodies (15). In the context of our hypothesis, a reduction in circulating CD56^dim^ NK cells might reflect homing of this cytotoxic subset into the rejecting allograft.

It is problematic as to whether human NK cell functions are adequately inhibited by current immunosuppression regimes. An *in vitro* study of peripheral blood NK cells from kidney transplant recipients demonstrated that immunosuppression did not affect the capacity of NK cells to respond to stimulation. In this study, circulating NK cells from recipients receiving immunosuppression secreted equivalent levels of IFN-γ, perforin, and granzyme A in response to stimulation with HLA class I-negative K562 cells compared with NK cells from healthy individuals (16). The inability of current immunosuppression regimes to downregulate activated NK cell functions represents a therapeutic opportunity. Novel treatments with the specificity to target either activated NK cells and/or their effector functions warrant testing.

In addition to our novel human NK cell subset data, our present study also examined the absolute numbers of other lymphocyte populations (e.g., B lymphocytes) in different patterns of immune-mediated rejection. Notably, we demonstrated significant increases in absolute numbers of B lymphocytes in TCMR biopsies (Figure 2B), but not in AMR biopsies (Figure 3B). Although an unexpected result given the role of B lymphocyte lineage cells (plasma cells) in antibody production, these findings are in fact consistent with previous reports of B lymphocyte infiltration significantly associating with TCMR, but not with AMR (32, 33). These analogous findings from these earlier IHC-based studies further confirm the efficiency and integrity of our multi-color flow cytometric approach.

In summary, we report the first comprehensive characterization of discrete kidney NK cell subsets in human allograft rejection. Our data provide the first evidence that human kidney NK cells may have subset-specific functional roles in the pathobiology of TCMR vs. AMR. Further evaluation of the kidney NK cell compartment in allograft models will test its utility in high precision tissue-based diagnostics. In addition, this will foster therapeutic approaches that specifically target the recruitment (e.g., chemokine receptors) or triggering (e.g., activating receptors) of discrete NK cell subsets dependent on the pathological conditions.

## Data Availability

All datasets generated for this study are included in the manuscript.

## Ethics Statement

Written informed consent for participation in the study was obtained. The Royal Brisbane and Women's Hospital Human Research Ethics Committee (2006/072) and the Princess Alexandra Hospital Ethics Committee (HREC/16/QPAH/214) approved the study.

## Author Contributions

Each author has participated sufficiently in the work to take public responsibility for the content. KK, RF, SH, GJ, JU, RW, AK, and HH conceived and designed the study. KK, SH, MH, KG, ES, BL, XW, and AK carried out experiments and analyzed the data. KK, SH, AK, and HH drafted the paper. All authors revised and approved the final version of the manuscript.

### Conflict of Interest Statement

The authors declare that the research was conducted in the absence of any commercial or financial relationships that could be construed as a potential conflict of interest.

## References

[1] Kloc M Ghobrial RM. Chronic allograft rejection: a significant hurdle to transplant success. Burns Trauma. (2014) 2:3–10. 10.4103/2321-3868.121646 27574640 PMC4994504

[2] Racusen LC Solez K Colvin RB Bonsib SM Castro MC Cavallo T. The Banff 97 working classification of renal allograft pathology. Kidney Int. (1999) 55:713–23. 10.1046/j.1523-1755.1999.00299.x 9987096

[3] Solez K Racusen LC. The Banff classification revisited. Kidney Int. (2013) 83:201–6. 10.1038/ki.2012.395 23235566

[4] Halloran PF Wadgymar A Ritchie S Falk J Solez K Srinivasa NS. The significance of the anti-class I antibody response. I. Clinical and pathologic features of anti-class I-mediated rejection. Transplantation. (1990) 49:85–91. 10.1097/00007890-199001000-00019 2301035

[5] Trpkov K Campbell P Pazderka F Cockfield S Solez K Halloran PF. Pathologic features of acute renal allograft rejection associated with donor-specific antibody, analysis using the Banff grading schema. Transplantation. (1996) 61:1586–92. 10.1097/00007890-199606150-00007 8669102

[6] Halloran PF. T cell-mediated rejection of kidney transplants: a personal viewpoint. Am J Transplant. (2010) 10:1126–34. 10.1111/j.1600-6143.2010.03053.x 20346061

[7] Spits H Di Santo JP. The expanding family of innate lymphoid cells: regulators and effectors of immunity and tissue remodeling. Nat Immunol. (2011) 12:21–7. 10.1038/ni.1962 21113163

[8] Angelo LS Banerjee PP Monaco-Shawver L Rosen JB Makedonas G Forbes LR. Practical NK cell phenotyping and variability in healthy adults. Immunol Res. (2015) 62:341–56. 10.1007/s12026-015-8664-y 26013798 PMC4470870

[9] Jacobs R Hintzen G Kemper A Beul K Kempf S Behrens G. CD56bright cells differ in their KIR repertoire and cytotoxic features from CD56dim NK cells. Eur J Immunol. (2001) 31:3121–6. 10.1002/1521-4141(2001010)31:10<3121::AID-IMMU3121>3.0.CO;2-4 11592089

[10] Zhang Z-X Huang X Jiang J Lau A Yin Z Liu W. Natural killer cells mediate long-term kidney allograft injury. Transplantation. (2015) 99:916–24. 10.1097/TP.0000000000000665 25719259

[11] Kohei N Tanaka T Tanabe K Masumori N Dvorina N Valujskikh A. Natural killer cells play a critical role in mediating inflammation and graft failure during antibody-mediated rejection of kidney allografts. Kidney Int. (2016) 89:1293–306. 10.1016/j.kint.2016.02.030 27165816 PMC4868788

[12] Yagisawa T Tanaka T Miyairi S Tanabe K Dvorina N Yokoyama WM In the absence of natural killer cell activation donor-specific antibody mediates chronic, but not acute, kidney allograft rejection. Kidney Int. (2019) 95:350–62. 10.1016/j.kint.2018.08.041 30503624 PMC6342643

[13] Hidalgo L Sis B Sellares J Campbell P Mengel M Einecke G. NK cell transcripts and NK cells in kidney biopsies from patients with donor-specific antibodies: evidence for NK cell involvement in antibody-mediated rejection. Am J Transplant. (2010) 10:1812–22. 10.1111/j.1600-6143.2010.03201.x 20659089

[14] Hidalgo L Sellares J Sis B Mengel M Chang J Halloran P. Interpreting NK cell transcripts versus T cell transcripts in renal transplant biopsies. Am J Transplant. (2012) 12:1180–91. 10.1111/j.1600-6143.2011.03970.x 22390872

[15] Crespo M Yelamos J Redondo D Muntasell A Perez-Saéz M López-Montañés M. Circulating NK-cell subsets in renal allograft recipients with anti-HLA donor-specific antibodies. Am J Transplant. (2015) 15:806–14. 10.1111/ajt.13010 25656947

[16] Hoffmann U Neudörfl C Daemen K Keil J Stevanovic-Meyer M Lehner F. NK cells of kidney transplant recipients display an activated phenotype that is influenced by immunosuppression and pathological staging. PLoS ONE. (2015) 10:e0132484. 10.1371/journal.pone.0132484 26147651 PMC4492590

[17] Venner J Hidalgo L Famulski K Chang J Halloran P. The molecular landscape of antibody-mediated kidney transplant rejection: evidence for NK involvement through CD16a Fc receptors. Am J Transplant. (2015) 15:1336–48. 10.1111/ajt.13115 25787894

[18] Yazdani S Callemeyn J Gazut S Lerut E de Loor H Wevers M. Natural killer cell infiltration is discriminative for antibody-mediated rejection and predicts outcome after kidney transplantation. Kidney Int. (2018) 95:188–98. 10.1016/j.kint.2018.08.027 30396694

[19] Shin S Kim YH Cho YM Park Y Han S Choi BH. Interpreting CD56+ and CD163+ infiltrates in early versus late renal transplant biopsies. Am J Nephrol. (2015) 41:362–9. 10.1159/000430473 26087825

[20] dos Santos DC Campos EF Saraiva Câmara NO David DSR Malheiros DMAC. Compartment-specific expression of natural killer cell markers in renal transplantation: immune profile in acute rejection. Transpl Int. (2016) 29:443–52. 10.1111/tri.12726 26615051

[21] Hirohashi T Chase C Della Pelle P Sebastian D Alessandrini A Madsen J. A novel pathway of chronic allograft rejection mediated by NK cells and alloantibody. Am J Transplant. (2012) 12:313–21. 10.1111/j.1600-6143.2011.03836.x 22070565 PMC3667648

[22] dos Santos DC Saraiva Camara NO David DSR Malheiros DMAC. Expression patterns of CD56+ and CD16+ cells in renal transplant biopsies with acute rejection: associations with microcirculation injuries and graft survival. Nephrology. (2017) 22:993–1001. 10.1111/nep.12897 27538059

[23] Haas M Sis B Racusen LC Solez K Glotz D Colvin RB. Banff 2013 meeting report: inclusion of C4d-negative antibody-mediated rejection and antibody-associated arterial lesions. Am J Transplant. (2014) 14:272–83. 10.1111/ajt.12590 24472190

[24] Kildey K Law BMP Muczynski KA Wilkinson R Healy H Kassianos AJ Identification and quantitation of leukocyte populations in human kidney tissue by multi-parameter flow cytometry. Bioprotocol. (2018) 8:e2980 10.21769/BioProtoc.2980 PMC8328643 34395780

[25] Muczynski KA Leca N Anderson AE Kieran N Anderson SK. Multicolor flow cytometry and cytokine analysis provides enhanced information on kidney transplant biopsies. Kidney Int Rep. (2018) 3:956–69. 10.1016/j.ekir.2018.02.012 29989006 PMC6035131

[26] Hadad U Martinez O Krams SM NK cells after transplantation: friend or foe. Immunol Res. (2014) 58:259–67. 10.1007/s12026-014-8493-4 24522700

[27] Law BM Wilkinson R Wang X Kildey K Lindner M Rist MJ. Interferon-γ production by tubulointerstitial human CD56bright natural killer cells contributes to renal fibrosis and chronic kidney disease progression. Kidney Int. (2017) 92:79–88. 10.1016/j.kint.2017.02.006 28396119

[28] Hancock WW Gao W Faia KL Csizmadia V. Chemokines and their receptors in allograft rejection. Curr Opin Immunol. (2000) 12:511–6. 10.1016/S0952-7915(00)00130-8 11007352

[29] Martín-Fontecha A Thomsen LL Brett S Gerard C Lipp M Lanzavecchia A. Induced recruitment of NK cells to lymph nodes provides IFN-γ for T H 1 priming. Nat Immunol. (2004) 5:1260. 10.1038/ni1138 15531883

[30] Racusen LC Colvin RB Solez K Mihatsch MJ Halloran PF Campbell PM. Antibody-mediated rejection criteria–an addition to the Banff′ 97 classification of renal allograft rejection. Am J Transplant. (2003) 3:708–14. 10.1034/j.1600-6143.2003.00072.x 12780562

[31] Neudoerfl C Mueller BJ Blume C Daemen K Stevanovic-Meyer M Keil J. The peripheral NK cell repertoire after kidney transplantation is modulated by different immunosuppressive drugs. Front Immunol. (2013) 4:46. 10.3389/fimmu.2013.00046 23450662 PMC3584176

[32] Tsai EW Rianthavorn P Gjertson DW Wallace WD Reed EF Ettenger RB. CD20+ lymphocytes in renal allografts are associated with poor graft survival in pediatric patients. Transplantation. (2006) 82:1769–73. 10.1097/01.tp.0000250572.46679.45 17198274

[33] Carpio VN Noronha Ide L Martins HL Jobim LF Gil BC Kulzer AS. Expression patterns of B cells in acute kidney transplant rejection. Exp Clin Transplant. (2014) 12:405–14. 10.6002/ect.2014.0049 25299368

